# Hinokiflavone resists HFD-induced obesity by promoting apoptosis in an IGF2BP2-mediated Bim m^6^A modification dependent manner

**DOI:** 10.1016/j.jbc.2024.107721

**Published:** 2024-08-29

**Authors:** Mingyu Wang, Mingkun Chao, Haozhe Han, Tiantian Zhao, Wenyong Yan, Gongshe Yang, Weijun Pang, Rui Cai

**Affiliations:** Laboratory of Animal Fat Deposition and Muscle Development, College of Animal Science and Technology, Northwest A&F University, Yangling, Shaanxi, China

**Keywords:** obesity, hinokiflavone, IGF2BP2, m^6^A modification, apoptosis

## Abstract

Obesity has emerged as a major health risk on a global scale. Hinokiflavone (HF), a natural small molecule, extracted from plants like cypress, exhibits diverse chemical structures and low synthesis costs. Using high-fat diet-induced obese mice models, we found that HF suppresses obesity by inducing apoptosis in adipose tissue. Adipocyte apoptosis helps maintain tissue health by removing aging, damaged, or excess cells in adipose tissue, which is crucial in preventing obesity and metabolic diseases. We found that HF can specifically bind to insulin-like growth factor 2 mRNA binding protein 2 to promote the stability of N6-methyladenosine-modified Bim, inducing mitochondrial outer membrane permeabilization. Mitochondrial outer membrane permeabilization leads to Caspase9/3-mediated adipocyte mitochondrial apoptosis, alleviating obesity induced by a high-fat diet. The proapoptotic effect of HF offers a controlled means for weight loss. This study reveals the potential of small molecule HF in developing new therapeutic approaches in drug development and biomedical research.

Obesity has become a global public health problem (1, 2). According to reports, in 2016, about 39% of adults globally had a body mass index (BMI) of more than 25 kg/m^2^, which was defined as overweight; while about 12% of adults had a BMI of more than 30, which was defined as obesity (3). It is estimated that by 2025, nearly 20% of the world's adults may be obese (4). Compared with normal weight, obesity is associated with an increased risk of many chronic diseases, such as cardiovascular disease, type 2 diabetes, hypertension, and certain cancers (5, 6, 7). Obesity is accompanied by the remodeling of white adipose tissue (WAT), which serves as a major endocrine and lipid storage organ. WAT is mainly composed of preadipocytes and adipocytes, as well as immune cells such as macrophages, which jointly maintain and monitor the integrity, metabolic function, and hormone sensitivity of adipocytes (8).

WAT exhibits plasticity in adapting to different nutritional and metabolic states through precisely regulating proliferation and apoptosis (9, 10). Adipocyte apoptosis is a programmed cell death way that maintains tissue health by removing aging, damaged, or redundant cells and plays an important role in preventing obesity and metabolic diseases (11, 12). Intrinsic apoptosis of adipocyte starts with an imbalance between proapoptotic proteins (Bim and Bid) and antiapoptotic proteins (Bcl-2, Bcl-xL, and Mcl-1). This imbalance favors the proapoptotic proteins, leading to the formation of pores by Bax/Bak in the mitochondrial outer membrane (13). This process is a defining hallmark of mitochondrial apoptosis and is known as mitochondrial outer membrane permeabilization (MOMP) (14). Subsequently, a cascade reaction of caspases 9 and 3 will be triggered to execute cell apoptosis (15, 16).

Insulin-like growth factor 2 mRNA binding protein 2 (IGF2BP2) is an N6-methyladenosine (m^6^A) reader with a molecular mass of 66 kDa. It contains two N-terminal RNA-recognition motifs and four C-terminal human heterogeneous nuclear ribonucleoprotein-K homology domains (17). These structural domains are important for the binding and recognition of RNA molecules in posttranscriptional gene regulation (18, 19). By interacting with the untranslated regions of mRNA molecules, IGF2BP2 can influence mRNA stability, transport, and translation (20). In fact, IGF2BP2 plays an important role in the occurrence of metabolic diseases such as obesity, T2D, and cancers (21, 22). Recently, it has been demonstrated that IGF2BP2 reads m^6^A, the most abundant internal RNA modification in eukaryotic cells (23, 24). By binding to m^6^A-modified RNAs, IGF2BP2 enhances RNA stability and translation (25, 26).

Recently, an increasing number of bioactive phytochemicals have been used in the discovery and prediction of drugs for the treatment of obesity. Research has found that the intake of polyphenols, such as flavonoids, in the mediterranean diet is associated with a decrease in waist-to-hip circumference (4). The global average intake of flavonoids ranges from 150 to 600 mg per day. However, there is considerable variation between countries (27). In the United States, the consumption of flavonoids is about 190 mg per day, while in Spain it is about 313 mg per day, and in Australia it is about 454 mg per day (28, 29). Therefore, there is an urgent need to explore the mechanisms of action of flavonoid compounds in order to provide targets for the treatment of obesity. Hinokiflavone (HF) is a small molecule belonging to the flavonoid class, which possesses broad biological activity. It has been reported to have a wide range of pharmacological activities, including metabolism regulation, anticancer, and antibacterial functions (30, 31).

In this study, we found that HF resists high-fat diet (HFD)-induced obesity by binding to IGF2BP2 and promoting Bim m^6^A modification-mediated apoptosis in adipocytes.

## Results

### The improved metabolic profile is observed in HF-treated HFD mice

**Figure 1:**
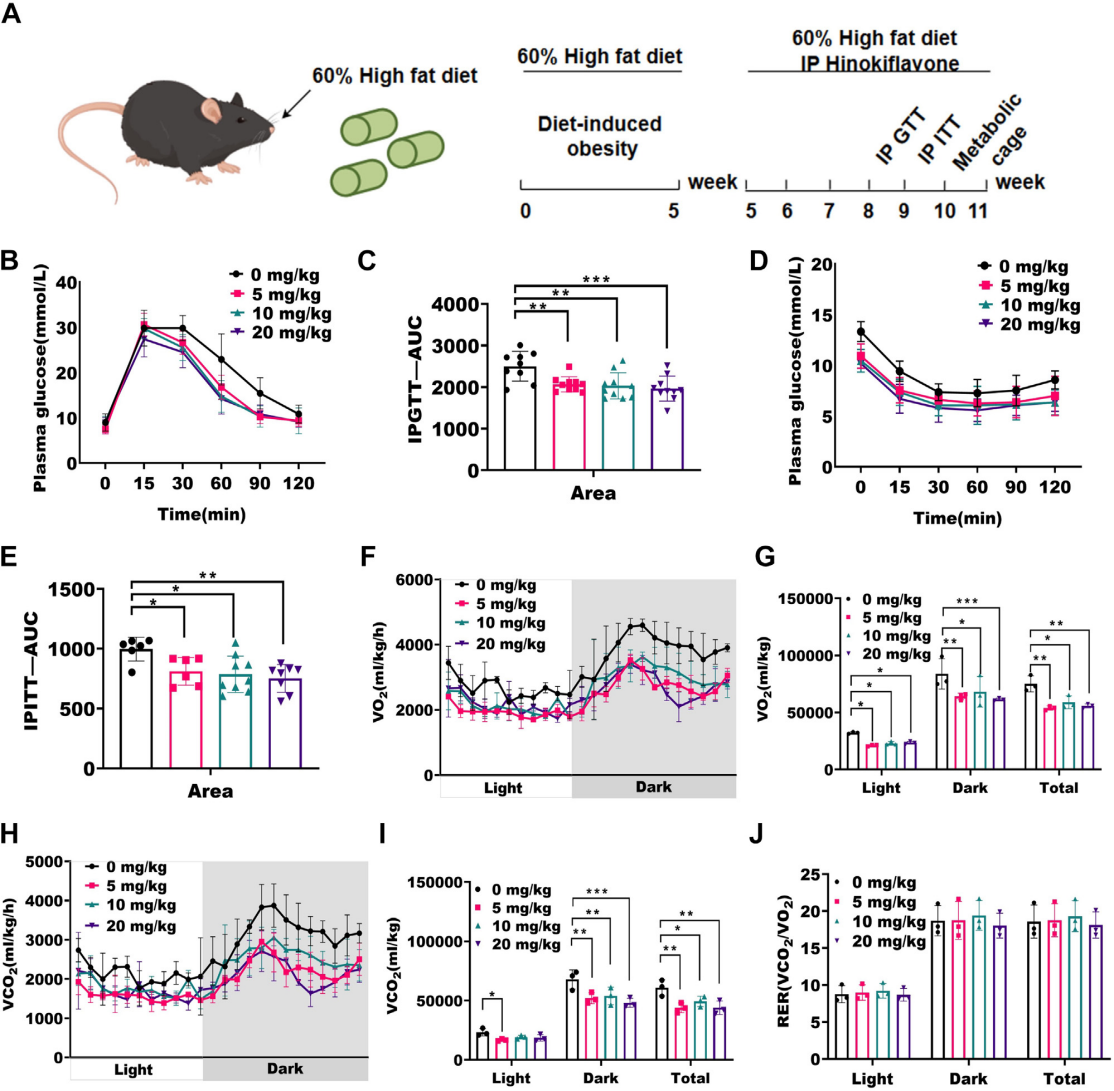
**The improved metabolic profile is observed in HF-treated HFD mice.***A*, C57BL/6 mice were induced obesity with a 60% high-fat diet, treated with HF by intraperitoneal injection, conducted IPGTT in the eighth week of feeding, IPITT in the ninth week, and metabolic cage experiments in the 10th week. *B*, graph of plasma glucose concentration in GTT experiment. *C*, AUC graph in GTT experiment, (n = 9–10), ∗∗*p* < 0.01, ∗∗∗*p* < 0.001 by One Way ANOVA between paired groups. *D*, graph of plasma glucose concentration in ITT experiment. *E*, AUC graph in ITT experiment, (n = 6–9), ∗*p* < 0.05, ∗∗*p* < 0.01 by One Way ANOVA between paired groups. *F*, measurement of oxygen consumption in mice during the light (8:00–20:00) and night (20:00–8:00). *G*, AUC graph in oxygen consumption, (n = 3), ∗*p* < 0.05, ∗∗*p* < 0.01, ∗∗∗*p* < 0.001 by One Way ANOVA between paired groups. *H*, measurement of carbon dioxide production in mice during the light (8:00–20:00) and night (20:00–8:00). *I*, AUC graph in carbon dioxide production, (n = 3), ∗*p* < 0.05, ∗∗*p* < 0.01, ∗∗∗*p* < 0.001 by one way ANOVA between paired groups. *J*, AUC graph of RER changes in mice during the light (8:00–20:00) and night (20:00–8:00) in metabolic cage experiments, (n = 3). Data are expressed as means ± SD. AUC, area under the curve; GTT, glucose tolerance test; HF, hinokiflavone; HFD, high-fat diet; IP, intraperitoneal; ITT, insulin tolerance test; RER, respiratory exchange ratio.

We examined the effect of HF on obesity by feeding control and HF intraperitoneally injected (HF-IP) mice with HFD containing 60% fat for 11 weeks (Fig. 1*A*). When feeding mice with HFD until reaching an average weight of 33 g (at 8 weeks of age), we administered HF. Based on existing research, we studied three concentrations of 5 mg/kg, 10 mg/kg, and 20 mg/kg in mice (31, 32, 33). HF-IP HFD mice demonstrated improved glucose tolerance compared to HFD mice, as indicated by the decreased area under the curve in the glucose tolerance test (GTT) (Fig. 1, *B* and *C*). The insulin tolerance test (ITT) revealed that HF-IP HFD mice improved insulin sensitivity (Fig. 1, *D* and *E*). HF enhanced the mice blood glucose metabolism capability. Compared to HFD mice, HF-IP HFD mice exhibited lower basal metabolic rate. HF-IP HFD mice there was a significant decrease in VO_2_, indicating a lower O_2_ consumption rate in mice (Fig. 1, *F* and *G*). Consistently, there was also a significant decrease in VCO_2_, suggesting a reduction in CO_2_ production in mice (Fig. 1, *H* and *I*). Respiratory exchange ratio (RER) showed no difference, indicating that the energy source of the mice did not change significantly (Fig. 1*J*). These results suggest that HF improved glucose tolerance and insulin sensitivity in HFD-induced obese mice.

### HF is resistant to HFD-induced obesity in mice

**Figure 2:**
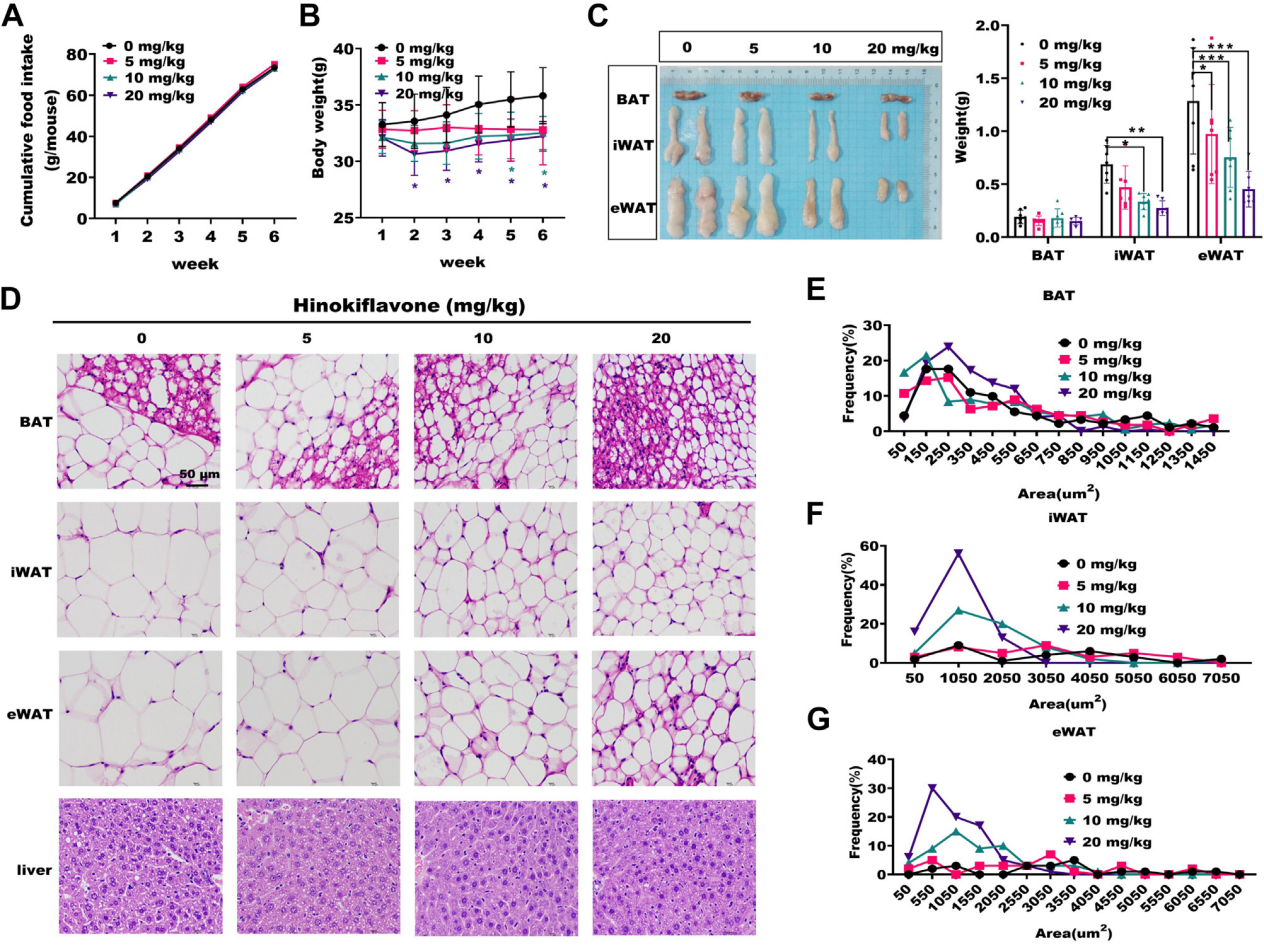
**HF is resistant to HFD-induced obesity in mice.***A*, cumulative food intake statistics during HF-IP in HFD mice. *B*, body weight change statistics in HFD mice during HF-IP during 6 weeks, (n = 10), ∗*p* < 0.05 by one way ANOVA between paired groups. *C*, fat tissue weight statistics in HFD mice, (n = 7), ∗*p* < 0.05, ∗∗*p* < 0.01, ∗∗∗*p* < 0.001 by one way ANOVA between paired groups. *D*, HFD mice's adipose tissue HE stained sections. The scale bars represent 50 μm. *E*, *brown* adipocyte area statistics in HFD mice. *F*, cellular area statistics of inguinal WAT in HFD mice. *G*, cellular area statistics of epididymal WAT in HFD mice. Data are expressed as means ± SD. BAT, brown adipose tissue; eWAT, epididymal white adipose tissue; H&E, hematoxylin-eosin; HF, Hinokiflavone; HFD, high-fat diet; HF-IP, HF intraperitoneally injected; iWAT, inguinal white adipose tissue.

**Supplemental Figure 1:**
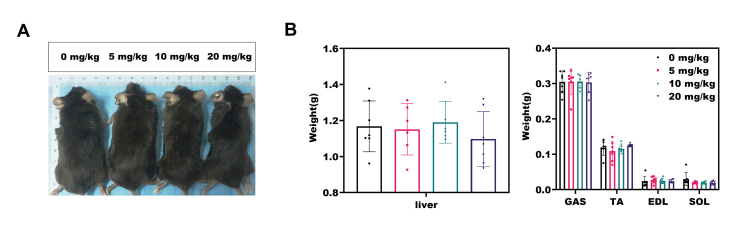


Under HFD feeding conditions, no differences were observed in food intake between HFD and HF-IP HFD mice (Fig. 2*A*). HF-IP mice exhibited a significant decrease in body weight and changes in morphology (Figs. 2*B* and S1*A*). This explained the decrease in VO_2_ and VCO_2_ in HF-IP HFD mice in Figure 1. The decrease in body weight led to a lower basal metabolic rate, resulting in reduced oxygen consumption and carbon dioxide production, while RER showed no significant change. Additionally, we observed a significant reduction in mouse adipose tissue weight, indicating that HF is beneficial for reducing obesity (Fig. 2*C*). HE staining showed a significant decrease in the adipocyte area of inguinal white adipose tissue and epididymal white adipose tissue, especially pronounced in 20 mg/kg HF-IP mice (Fig. 2, *D*–*G*). The decrease of adipocyte area in brown adipose tissue was not significant, consistent with no change in brown adipose tissue weight. Fig. S1*B* showed no significant decrease in the weights of muscle tissue and liver. These results indicate that HF significantly reduced the weight and adipocyte area of WAT, leading to weight loss in obese mice.

### Substantial apoptosis of adipose tissue occurs in HF-IP obese mice

**Figure 3:**
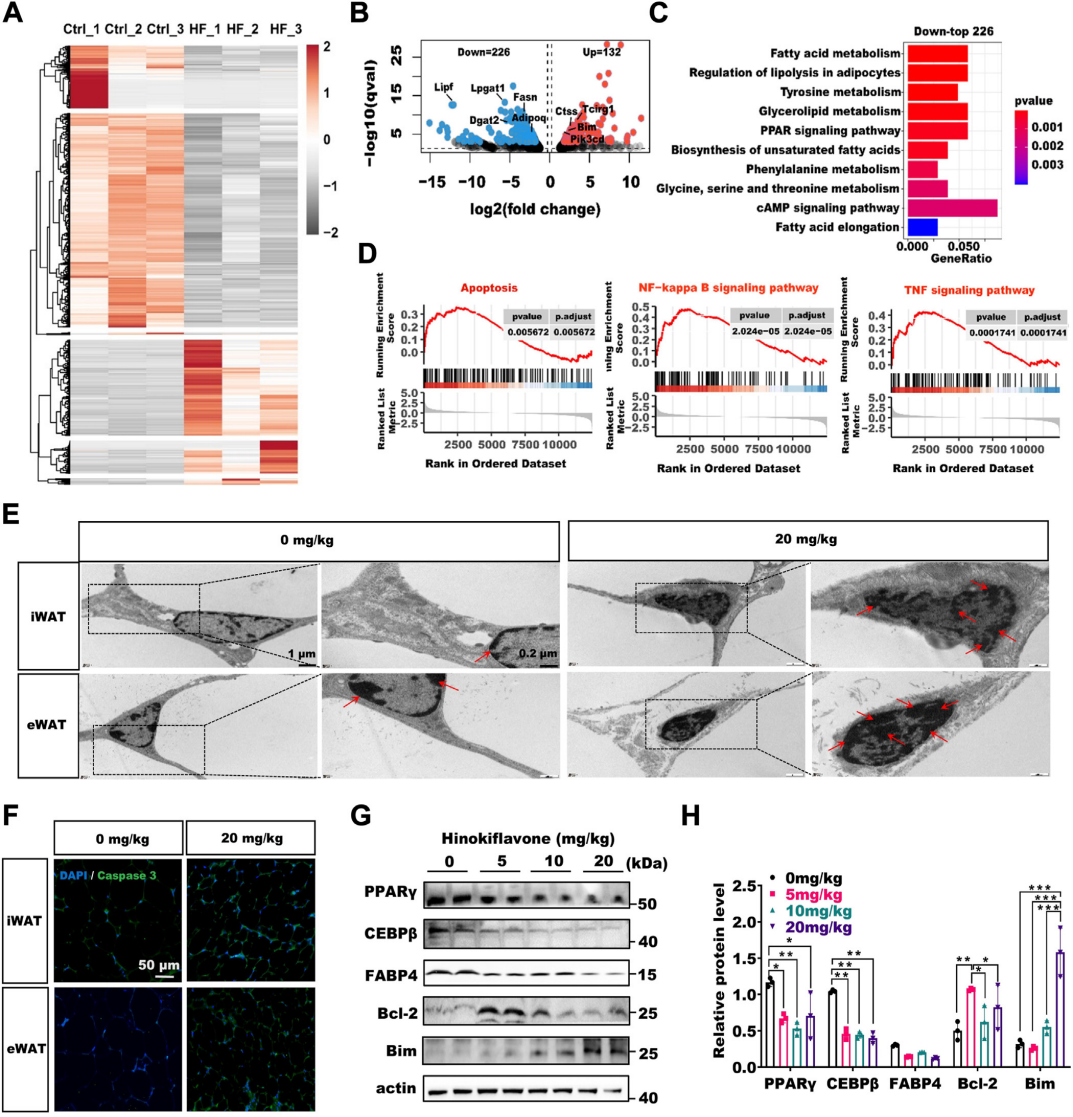
**Substantial apoptosis of adipose tissue occurs in HF-IP obese mice.***A*, heatmap analysis of RNA-seq data from WAT in HF-IP mice and control mice. *B*, Volcano plot analysis of RNA-seq data (q value < 0.001). *C*, KEGG enrichment analysis of downregulated genes. *D*, GSEA of genes related to Apoptosis, NF-κB, and TNF signaling pathways in upregulated genes. *E*, cell morphology of adipose tissue captured by electron microscopy. The scale bars represent 0.2 μm and 1 μm respectively. *F*, immunofluorescent staining of Caspase 3 and DAPI in adipose tissue. The scale bars represent 50 μm. *G*, the protein level of adipogenic and apoptotic genes in WAT of HFD mice treated with different HF concentrations. *H*, statistical analysis of protein expression in WAT, (n = 3), ∗*p* < 0.05, ∗∗*p* < 0.01, ∗∗∗*p* < 0.001 by Two Way ANOVA between paired groups. Data are expressed as means ± SD. DAPI, 4′,6-diamidino-2-phenylindole; eWAT, epididymal white adipose tissue; GSEA, Gene Set Enrichment Analysis; HF, Hinokiflavone; HFD, high-fat diet; HF-IP, HF intraperitoneally; iWAT, inguinal white adipose tissue; KEGG, Kyoto Encyclopedia of Genes and Genomes; TNF, tumor necrosis factor.

To explore the reasons of decrease in WAT caused by HF-IP, we performed transcriptome sequencing on WAT samples from 0 mg/kg and 20 mg/kg HF-IP mice. The heatmap depicted the differential gene expression profiles (Fig. 3*A*). A total of 358 genes showed significant change, with 226 genes downregulated and 132 genes upregulated. We observed a significant downregulation of adipose markers *Adipoq*, *Fasn*, *Dgat2*, *Lpgat1*, and *Lipf*, while the apoptosis gene *Bim* was significantly upregulated (Fig. 3*B*). Pathways related to fatty acid metabolism, peroxisome proliferator-activated receptor-γ signaling and others also exhibited significant downregulation, suggesting that the decrease in adipogenesis may be attributed to apoptosis (Fig. 3*C*). We hypothesized that HF may exert its effects through apoptosis, so we examined the apoptosis status of adipose tissue. Subsequently, Gene Set Enrichment Analysis revealed a significant upregulation of gene sets associated with apoptosis, tumor necrosis factor, and nuclear factor kappaB (NF-κB) signaling pathways in HF-IP HFD mice (Fig. 3*D*). Studies have shown that tumor necrosis factor can induce the formation of the death-inducing complex leading to apoptosis, while activation of NF-κB could prevent the formation of the death-inducing complex (12, 34). Therefore, we propose that the body undergoes mutual negative regulation between apoptosis and antiapoptotic mechanisms, inevitably resulting in the reduction of adipose tissue.

To validate our hypothesis, we conducted investigations on apoptosis-related biological phenomena and marker genes. Interestingly, in the WAT of HF-IP, transmission electron microscopy results revealed significant chromatin condensation in the nuclei of adipocytes, consistent with the biological characteristics of apoptosis (Fig. 3*E*). Furthermore, immunofluorescence staining of Caspase 3 in WAT showed substantial Caspase 3 aggregation (Fig. 3*F*). Additionally, we performed Western blot analysis on adipogenic marker genes and other apoptosis-related genes, *Bcl-2* (resistance to Bim protein) and *Bim*, which demonstrated a significant decrease in adipogenic proteins with an increase in apoptosis (Fig. 3, *G* and *H*). These results demonstrated that HF induced apoptosis in adipose tissue, leading to a reduced expression of key genes associated with adipogenesis in mice.

### HF activates the mitochondrial pathway of apoptosis

**Figure 4:**
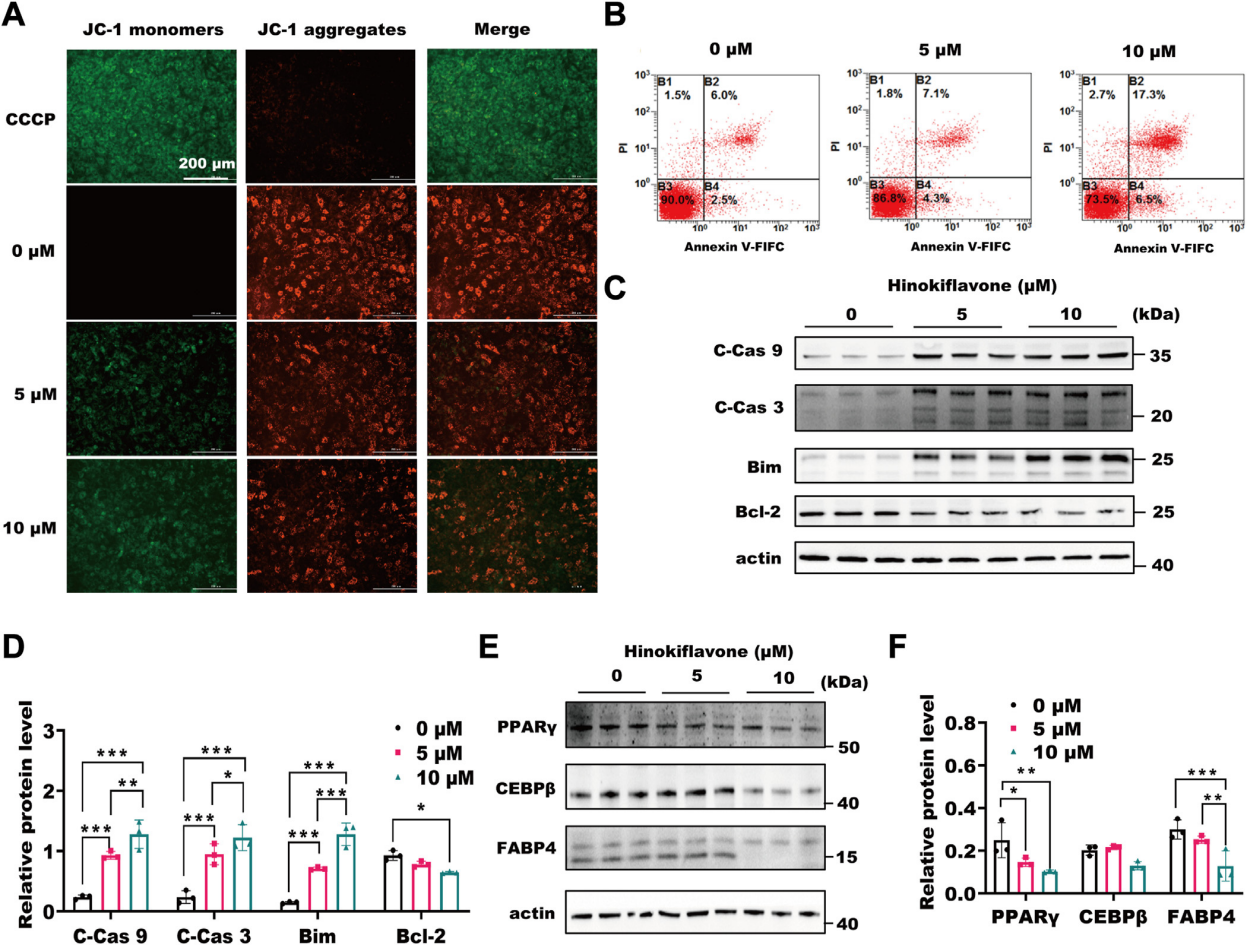
**HF activates the mitochondrial pathway of apoptosis.***A*, JC-1 fluorescence staining of adipogenic 3T3-L1 cells on the fourth day of differentiation. The scale bars represent 200 μm. *B*, flow cytometry analysis of adipogenic 3T3-L1 cells on the fourth day of differentiation by annexin V binding and propidium iodide (PI) uptake. *C*, the protein level of apoptotic genes in 3T3-L1 cells treated with different HF concentrations on the fourth day of differentiation. *D*, statistical analysis of apoptosis-protein expression, (n = 3), ∗*p* < 0.05, ∗∗*p* < 0.01, ∗∗∗*p* < 0.001 by Two Way ANOVA between paired groups. *E*, the protein level of adipogenic genes in 3T3-L1 cells treated with different HF concentrations on the fourth day of differentiation. *F*, statistical analysis of adipogenesis-related protein expression, (n = 3), ∗*p* < 0.05, ∗∗*p* < 0.01, ∗∗∗*p* < 0.001 by Two Way ANOVA between paired groups. Data are expressed as means ± SD. HF, Hinokiflavone

**Supplemental Figure 2:**
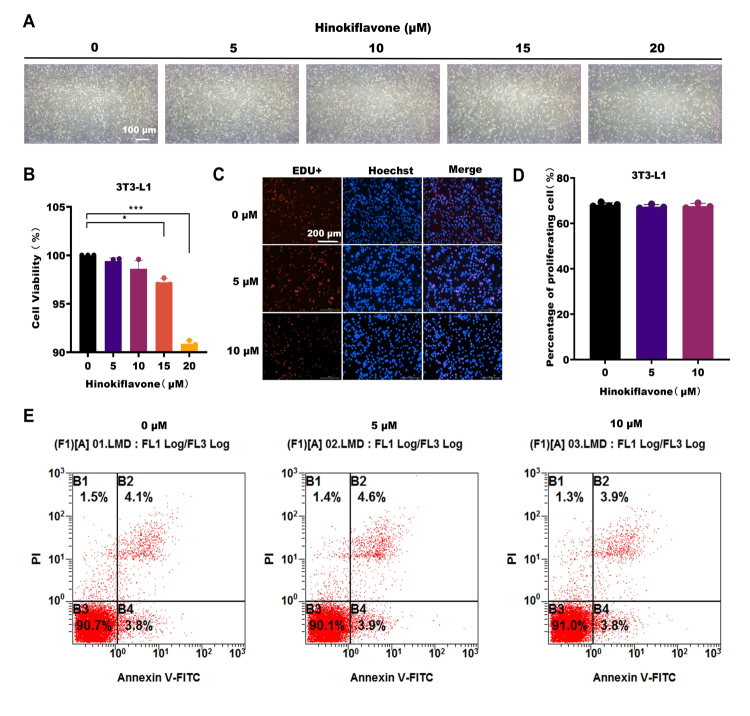


We simulated the effects of HF-IP on 3T3-L1 cells, and the results of Cell Counting Kit-8 and 5-ethynyl-2′-deoxyuridine (EDU) assays showed that concentrations below 10 μM were nontoxic to the cells (Fig. S2, *A–D*). Meanwhile, we examined the cell apoptosis during the proliferation process and found no apoptosis (Fig. S2*E*). We believe that the proliferating 3T3-L1 cells are fibroblasts and require induction of adipogenic differentiation to mimic adipose tissue apoptosis in mice. To assess whether HF treatment induces apoptosis in differentiated 3T3-L1 cells, we performed JC-1 staining, revealing MOMP, a hallmark characteristic of apoptosis (Fig. 4*A*). Flow cytometry analysis further confirmed that HF led to apoptosis in differentiated 3T3-L1 cells by annexin V binding and propidium iodide uptake (Fig. 4*B*). Similarly, the protein level of proapoptotic genes, *Cleaved (C)-Caspase 3*, *C-Caspase 9*, and *Bim*, were increased, antiapoptotic proteins Bcl-2 was decreased. However, adipogenic genes *PPARγ*, *CEBPβ*, and *FABP4* showed decreased protein levels (Fig. 4, *C*–*F*). These results demonstrate that HF induces apoptosis in differentiated 3T3-L1 cells.

### HF targets IGF2BP2 and influences its function

**Figure 5:**
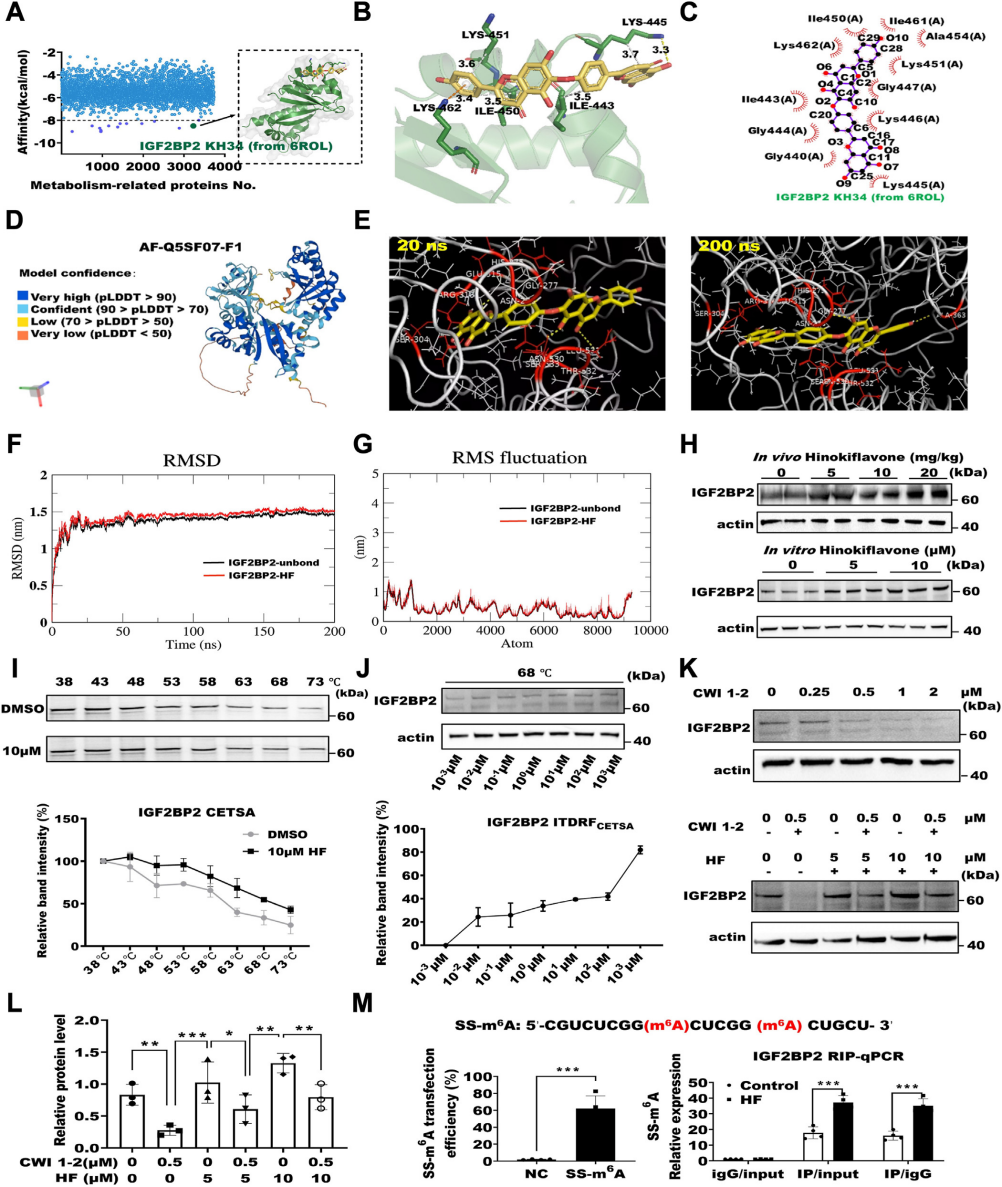
**HF targets IGF2BP2 and influences its function.***A*, virtual screening of metabolism-related proteins for natural small molecule HF. *B*, 3D visualization of molecular docking results of HF with IGF2BP2 KH34. *C*, 2D visualization of molecular docking results of HF with IGF2BP2 KH34. *D*, structure of IGF2BP2 built by AlphaFold2. *E*, conformation of binding between HF and IGF2BP2 during molecular dynamics simulation at 20 ns and 200 ns. *F*, RMSD plot of molecular dynamics simulation. *G*, RMSF plot of molecular dynamics simulation. *H*, Western blot analysis of IGF2BP2 protein expression in mice and 3T3-L1 cells treated with HF on the fourth day of differentiation. *I*, CETSA experiment to examine the effect of HF on the stability of IGF2BP2 protein during thermal denaturation. *J*, ITDRF_CETSA_ experiment to examine the effect of different concentrations of HF on the stability of IGF2BP2 protein during thermal denaturation at 68 °C. *K*, effect of cotreatment of specific inhibitor CWI1-2 of IGF2BP2 with HF on the expression level of IGF2BP2 detected by Western blot on the sixth day of differentiation. *L*, statistical analysis of IGF2BP2 protein expression, (n = 3), ∗*p* < 0.05, ∗∗*p* < 0.01, ∗∗∗*p* < 0.001 by two way ANOVA between paired groups. *M*, RIP-qPCR assay to detect the effect of HF on the binding ability of IGF2BP2 to m^6^A-modified RNA, (n = 4), ∗∗∗*p* < 0.001 by Student’s *t* test. Data are expressed as means ± SD. CETSA, cellular thermal shift assay; HF, hinokiflavone; IGF2BP2, insulin-like growth factor 2 mRNA binding protein 2; ITDRF_CETSA_, isothermal dose-response fingerprint CETSA; qPCR, quantitative real-time PCR; RIP, RNA immunoprecipitation; RMSF, root mean square fluctuation; SS-m^6^A, single-stranded RNA with m^6^A modification.

Apoptosis is a programmed cell death process closely related to cellular metabolism (35, 36, 37). To investigate how HF induces apoptosis, a metabolic phenomenon, we conducted virtual screening of proteins. By screening 3800 metabolism-related proteins against HF, we identified 14 proteins with an affinity of −8 kcal/mol or below (Fig. 5*A*). Previous research indicated that IGF2BP2 is a recognition protein that can enhance the stability of m^6^A-modified RNA. Besides, there are studies suggesting that IGF2BP2 is associated with apoptosis (38, 39, 40). To further investigate the binding between HF and IGF2BP2, we hypothesized that IGF2BP2 serves as an intermediary for regulatory function of HF. Using PyMOL for 3D visualization, we observed hydrogen bonds (yellow dashed lines), π-π interactions (orange dashed lines), and hydrophobic interactions (white dashed lines) between HF and IGF2BP2 (Fig. 5*B*). Using ligplot + for 2D visualization also revealed similar intermolecular forces (Fig. 5*C*). Interestingly, compared to the 3D visualization, the 2D visualization showed more hydrogen bonding interactions. We considered it is necessary to study the overall structure of IGF2BP2 and its binding process with HF. Therefore, we used AlphaFold2 (https://alphafold.com) to construct the structure of IGF2BP2 (Fig. 5*D*). Molecular dynamics simulations showed that from 20 ns to 200 ns, HF and IGF2BP2 remained bound (Fig. 5*E* and Movie S1). RMSD and root mean square fluctuation also exhibited good similarity and stability (Fig. 5, *F* and *G*), indicating the credibility of their binding.

We tested samples treated with HF *in vivo* and *in vitro*, and found that with increasing HF concentration, IGF2BP2 showed significant upregulation (Fig. 5*H*). To verify if HF enhanced its expression by binding to IGF2BP2, we conducted a cellular thermal shift assay (CETSA) experiment. It was worth noting that we used HF-treated cell lysates instead of directly treating cells to avoid false positives due to HF affecting IGF2BP2 expression through signaling pathways. Figure 5*I* showed that HF binding to IGF2BP2 alleviated the degradation of IGF2BP2 caused by temperature increase. Under 68 °C conditions, with the rise in HF concentration, the stability of IGF2BP2 improved, suggesting that HF's capacity to boost the stability of IGF2BP2 depended on concentration (Fig. 5*J*). This suggests that the binding of HF to IGF2BP2 enhanced the stability of IGF2BP2, leading to an increase in its expression levels.

Furthermore, we used the specific inhibitor of IGF2BP2, CWI1-2, and found that 5 μM and 10 μM of HF relieved the inhibitory effect of CWI1-2, suggesting that HF could act as an activator of IGF2BP2 (26) (Fig. 5, *K* and *L*). To investigate whether HF binding to IGF2BP2 affected its function in binding m^6^A-modified RNA, we synthesized single-stranded RNA with m^6^A modification (SS-m^6^A) and transfected differentiated 3T3-L1 cells. The results showed that HF treatment enhanced IGF2BP2's ability to bind SS-m^6^A (Fig. 5*M*). These results indicated that HF enhanced the levels of IGF2BP2 and its function by stabilizing m^6^A-modified RNA.

### IGF2BP2 regulates adipocyte apoptosis through m^6^A-modified Bim

**Figure 6:**
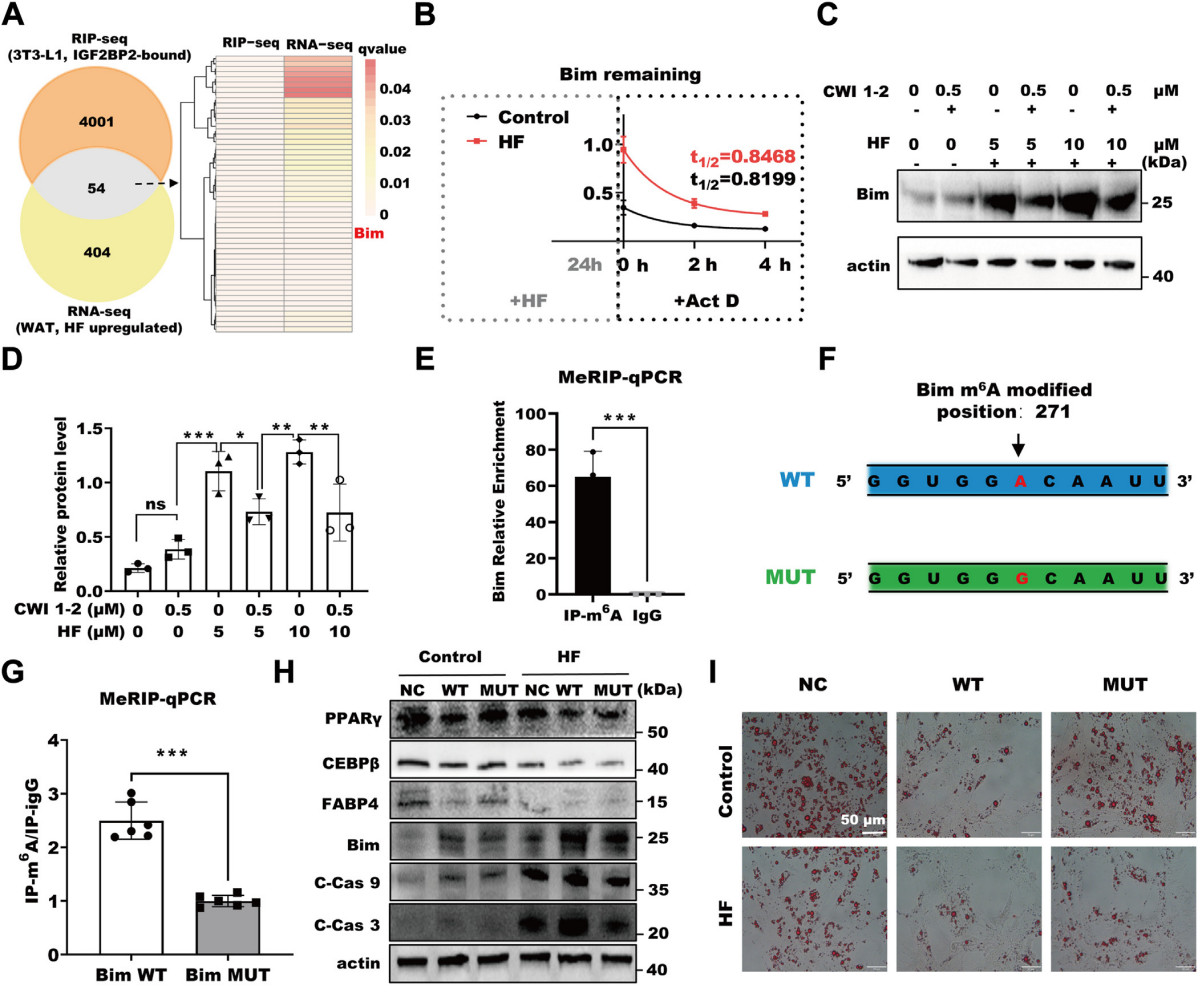
**IGF2BP2 regulates adipocyte apoptosis through m**^**6**^**A-modified Bim.***A*, intersection of IGF2BP2 RIP-seq data and upregulated RNA-seq data, with significance level indicated by q value. *B*, the effect of HF on the expression level and half-life of Bim mRNA. *C*, effect of cotreatment of specific inhibitor CWI1-2 of IGF2BP2 with HF on Bim expression detected by Western blot on the sixth day of differentiation. *D*, statistical analysis of Bim protein expression, (n = 3), ∗*p* < 0.05, ∗∗*p* < 0.01, ∗∗∗*p* < 0.001, ns non-significant by two way ANOVA between paired groups. *E*, MeRIP-qPCR assay to detect m^6^A modification on Bim mRNA, (n = 3), ∗∗∗*p* < 0.001 by Student’s *t* test. *F*, site-directed mutagenesis of the Bim plasmid. *G*, MeRIP-qPCR assay to examine m^6^A modification levels on Bim after mutation, (n = 6), ∗∗∗*p* < 0.001 by Student’s *t* test. *H*, effect of Bim mutation on the expression levels of adipogenesis and apoptosis-related proteins detected by Western blot on the sixth day of differentiation. *I*, Oil Red O staining to assess the effect of Bim mutation on cellular lipid deposition on the sixth day of differentiation. The scale bars represent 50 μm. Data are expressed as means ± SD. HF, hinokiflavone; IGF2BP2, insulin-like growth factor 2 mRNA binding protein 2; m^6^A, N6-methyladenosine; MeRIP, methylated RNA immunoprecipitation; qPCR, quantitative real-time PCR; RIP, RNA immunoprecipitation.

**Supplemental Figure 3:**
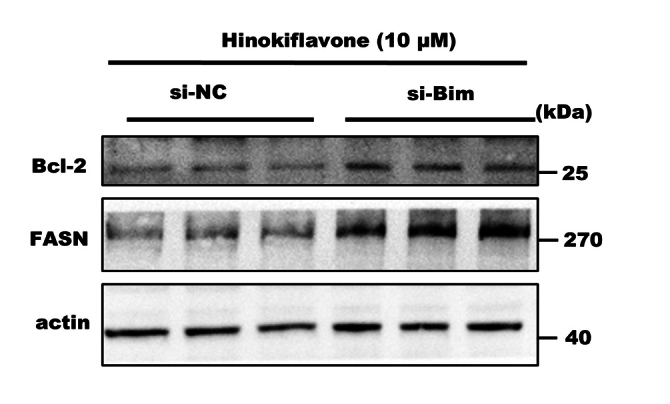


**Supplemental Figure 4:**
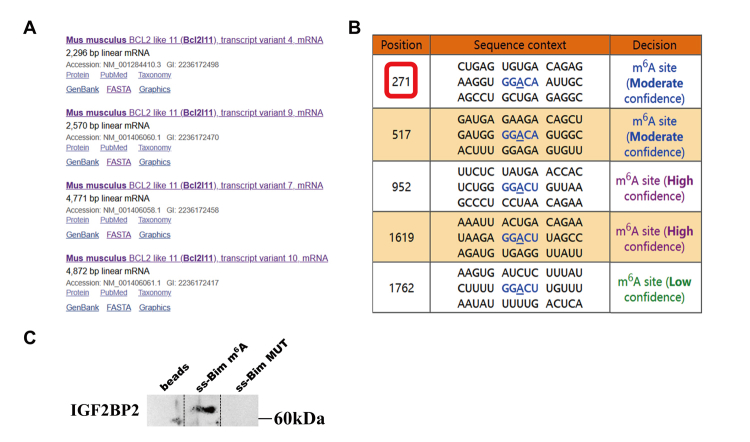


To investigate the downstream effects of IGF2BP2, we combined RNA immunoprecipitation (RIP)-seq and RNA-seq data and identified 54 overlapping genes, including the proapoptotic gene *Bim*, which is not only enriched in apoptosis signaling pathways, but also has m^6^A modification sites in the transcript (Figs. 6*A* and S4*B*). Examination of Bim mRNA showed that a 24 h treatment with HF increased Bim mRNA expression (Fig. 6*B*). Additionally, Act D treatment showed that HF extended the half-life of Bim mRNA, suggesting that HF enhanced mRNA stability bound by IGF2BP2 (Fig. 6*B*). Moreover, a *Bim* knockdown experiment was performed, and the results showed that the knockout of *Bim* significantly affected the expression of apoptosis and adipogenesis genes (Fig. S3). HF alleviated the inhibition of IGF2BP2 function by CWI1-2, resulting in increased Bim protein expression (Fig. 6, *C* and *D*). Through methylated RNA immunoprecipitation-quantitative real-time PCR, we observed m^6^A methylation modifications on Bim mRNA, indicating IGF2BP2 may function as a m^6^A reader (Fig. 6*E*). To investigate whether m^6^A modification of Bim is necessary in this process, we designed A271G mutant plasmids at conserved positions in different Bim transcripts (Figs. 6*F* and S4, *A* and *B*). Subsequent results demonstrated a significant decrease in m^6^A modifications on Bim MUT (Fig. 6*G*). Furthermore, we performed RNA-pull-down experiment to confirm that interaction between IGF2BP2 and Bim transcripts. The results showed IGF2BP2 can be detected in ss-Bi*m* m^6^A group, while not detected in ss-Bim MUT group (Fig. S4*C*). The results indicated that IGF2BP2 physically binds and recognizes Bim transcripts in an m^6^A-dependent manner. To investigate the impact of mutated m^6^A modification sites on HF-induced apoptosis, we transfected adipogenic differentiated 3T3-L1 cells. Notably, regardless of HF presence, apoptosis was attenuated in 3T3-L1 cells transfected Bim MUT, promoting adipogenesis (Fig. 6, *H* and *I*). This indicated that HF exerts proapoptotic and antiadipogenic functions through IGF2BP2 recognition of the 271th m^6^A site on Bim. Therefore, IGF2BP2 promotes adipocyte apoptosis through m^6^A-modified *Bim*.

## Discussion

In this study, we found that HF acts as a natural small molecule for resisting HFD-induced obesity. The principal mechanism is that HF targets IGF2BP2 and enhances stability of the m^6^A-modified Bim, leading to the occurrence of apoptosis in adipocytes. Natural small molecules are typically derived from organisms or natural resources such as plants, exhibiting high biocompatibility and bioactivity. It has been established that flavonoids have a significant impact in the mediterranean diet on reducing BMI and blood sugar levels (4, 41, 42). The basic structural feature of flavonoids is containing two benzene rings connected by three carbons, described as a "C6-C3-C6" linkage. HF is extracted from plants like cypress with two "C6-C3-C6" linkages. We wondered the effects and mechanisms of these molecules on obesity, so we used HFD-induced obese mice model. Interestingly, we found that HF suppressed obesity by inducing apoptosis in adipose tissue. It has been reported HF could induce apoptosis through the reactive oxygen species-mitochondrial apoptotic pathway in melanoma cells (43). Also, HF induces apoptosis *via* activating mitochondrial ROS/JNK/caspase pathway and inhibiting NF-κB activity in hepatocellular carcinoma (31). In this study, we report for the first time that HF inhibited adipogenesis by inducing apoptosis in adipocytes.

Adipocyte apoptosis is a spontaneous healthy process that regulates the number and size of cells, impacting fat metabolism and energy balance (44). In this study, we found that HF activated adipocyte apoptosis by mitochondrial pathway. It has been reported that mitochondrial apoptosis is an intrinsic pathway, regulated by members of the proapoptotic and antiapoptotic families (45). The BH3-only protein Bim can inhibit antiapoptotic members of the Bcl-2 family (such as Bcl-xL and Mcl-1) and activate Bak and Bax (13). It is worth noting that the HF led to apoptosis by increasing the protein level of proapoptotic genes *C-Caspase 3/9* and *Bim*, which is consistent with previous report that activation of Bax and Bak induces MOMP led to a gradual decrease in mitochondrial respiration and causing widespread mitochondrial dysfunction (14).

IGF2BP2, as an m^6^A reader, influences cell survival by enhancing the stability of m^6^A-modified mRNAs (25, 26). In this study, we found that HF could interact with KH34 of IGF2BP2 through hydrogen bonds, π-π and hydrophobic interactions, which is consistent with recent studies (18). Furthermore, molecular dynamics simulations demonstrated that IGF2BP2 could stably bind to HF, leading to increased expression levels and functionality. These results demonstrated that HF promoted the level of Bim through IGF2BP2. It is of interest to explore the apoptosis signaling pathway regulating the expression of adipogenic genes in further study.

In HFD mice, moderate apoptosis aids in controlling the amount of fat tissue and maintaining the stability of fat tissue. Therefore, some researchers have engineered transgenic mice that induce adipocyte apoptosis by targeting the activation of Caspase 8 (46). In this study, we found that HF could serve as an inducer of apoptosis in the adipose tissue of HFD mice, controlling the amount and metabolism of adipose tissue through the mitochondrial apoptosis pathway, providing a convenient method for reducing obesity. Early studies have reported that HF, as a MDM2 inhibitor, activates p53 signaling pathway to induce apoptosis in human colon cancer cells (47). Here, HF targets IGF2BP2 to enhance m^6^A-modified Bim expression, leading to a cascade reaction of Bim-MOMP-Caspase 9/3.

Intriguingly, we only observed significant weight loss in adipose tissue, with no abnormalities in muscle tissues and liver. This could be due to the slower metabolic rate of fat tissue, as well as HF's lipid-soluble nature, making it easier to enter fat tissue through the principle of similar solubility.

Taken together, we found that HF promotes weight loss through IGF2BP2-medimated Bim m^6^A modification in HFD mice. The interesting discovery of the interaction of IGF2BP2 and Bim provides novel insights into the relationship between adipogenesis and apoptosis through m^6^A modification mechanism. Our results also demonstrate that HF, the natural small molecule, plays a crucial role in drug development and biomedical research, providing new directions for discovering new drug targets and obesity treatment.

## Experimental procedures

### Animals

Forty three-week-old male C57BL/6J mice were purchased from Xi'an Jiaotong University (Xi'an, China). During the experiment, the animals were housed in a dedicated vivarium with controlled temperature, humidity, and a 12-h light/dark cycle. All experiments were approved by ethics committee of animal welfare and health of Northwest A&F University (Shaanxi, China, No. NWAFU-2023–0611).

### High-fat diet feeding

The 60% high-fat diet was purchased from Beijing Huafukang Biotechnology Co, Ltd. To prevent fat oxidation and spoilage, the feed was replaced in its entirety on a daily basis. At 9 weeks of age and with an average body weight of 32.5 g, a randomly selected mouse was found to have a substantial fat pad.

### Intraperitoneal (IP) injection of HF

The experimental mice were randomly divided into the following four groups (10 mice in each group): control group, 5 mg/kg HF group, 10 mg/kg HF group, and 20 mg/kg HF group. HF was dissolved in dimethyl sulfoxide and injected intraperitoneally into the animals in the treatment group. The control group was given the amount of dimethyl sulfoxide as administered to the 20 mg/kg HF group (48, 49, 50). Diet-induced obese mice were administered intraperitoneally every other day.

### Glucose and insulin tolerance tests

The control group consisted of nine mice, and each of the three experimental groups consisted of ten mice. For GTT and ITT, the mice fasted for 16 h (from 17:00–9:00) before the experiment. They were then intraperitoneally injected with a 20% glucose solution, with a dosage of 2 g/kg. The insulin dosage was 0.5U/kg, prepared as a solution of 0.05U/ml using saline, and the interval between GTT and ITT was 1 week.

### Metabolic cage

During the ninth week of feeding, the whole-body energy expenditure was monitored using the Comprehensive Lab Animal Monitoring System (CLAMS, Columbus Instruments) through indirect calorimetry. Oxygen consumption (VO_2_), carbon dioxide production (VCO_2_), and the RER were measured every 5 min. Each mouse was given a 1-day adaptation period before the experiment (n = 3).

### H&E staining

The mouse tissues were fixed in 4% paraformaldehyde for 24 h. After dehydration and embedding steps, continuous sections were made. The sectioned specimens were then stained, mounted on slides, and subjected to microscopic examination.

### Oil Red O staining

The fresh tissues were flash-frozen in liquid nitrogen and stored in a −80 °C freezer. The tissues were then trimmed to a length of approximately 2 mm. A layer of optimal cutting temperature embedding compound was applied to the sample holder, followed by placing the trimmed tissues on top. Additional optimal cutting temperature was applied to completely cover the tissues, and then the sample was allowed to solidify in a temperature-controlled cryostat. Continuous sections were then made using the cryostat, with a thickness of 5 to 6 μm. After staining and mounting, the sections were finally examined under a microscope.

### RNA sequencing and bioinformatics analysis

Total RNA was extracted from the WAT of both the 0 mg/kg HF and 20 mg/kg HF groups of mice. Pair-end sequencing of the RNA was conducted using the Illumina platform (Illumina). After filtering the raw data, checking for sequencing errors, and examining the GC content distribution, clean reads were obtained for subsequent analysis. To calculate fold changes, the DESeq2 package in R was used. The *p* values were adjusted for multiple comparisons using the false discovery rate method, and genes with a q value < 0.05 were considered significantly upregulated or downregulated. The heatmap package in R (https://cran.r-project.org/web/packages/ggplot2/index.html) was used to generate a heatmap, while the ggplot2 package was used to generate a volcano plot (q value < 0.001). For Kyoto Encyclopedia of Genes and Genomes analysis of significantly differentially expressed genes, the clusterProfiler (https://bioconductor.org/packages/clusterProfiler/) and enrichplot (https://bioconductor.org/packages/enrichplot/) packages in R were used. Gene Set Enrichment Analysis was performed to further analyze differentially expressed genes with a q value < 0.05. Both upregulated and downregulated genes were categorized using the hallmark gene set.

### RNA immunoprecipitation and high-throughput sequencing and bioinformatics analysis

Magna RIP RNA-Binding Protein Immunoprecipitation Kit (Merck sigma) was used for IGF2BP2 RIP. The 3T3-L1 cells were lysed by RIP lysis buffer. Magnetic beads were coincubated with IGF2BP2 antibody (Thermo Fisher Scientific). Nucleic acid-protein immune complexes were collected by immunoprecipitation of lysates with incubated magnetic beads overnight at 4 °C. RNA on the protein A/G magnetic beads was collected and purified using proteinase K Buffer, and so on, and then subjected to RNA high-throughput sequencing and biosignature analysis studies.

### Transmission electron microscopy

Prefixed with a 3% glutaraldehyde, then the fresh WAT tissue was postfixed in 1% osmium tetroxide, dehydrated in series acetone, infiltrated in Epox 812 for a longer, and embedded. The semithin sections were stained with methylene blue and ultrathin sections were cut with diamond knife, stained with uranyl acetate and lead citrate. Sections were examined with JEM-1400-FLASH Transmission Electron Microscope.

### Immunofluorescence

The paraffin sections were dewaxed and washed in water. High temperature antigen repair was performed using antigen repair buffer for 1 h. The sections were then incubated with blocking buffer (PBS with 5% w/v bovine serum albumin and 5% v/v normal goat serum) at room temperature for 1 h, followed by overnight incubation at 4 °C. The sections were then incubated with secondary antibody and 4′,6-diamidino-2-phenylindole, and finally blocked for observation. For EDU (Thermo Fisher Scientific) staining and JC-1 (MCE) staining of 3T3-L1 cells, after treatment with hinokiflavone (MCE), the cells were incubated with EDU working solution, CCCP and JC-1 working solution. Nuclei were stained using Hoechst working solution (Thermo Fisher Scientific).

### Protein isolation and western blot analysis

Total protein was isolated from adipose tissue by dissection. For 3T3-L1 cells total protein extraction was performed using a cell scraper. Total protein was homogenized for 30 min on ice using a lysate containing protease inhibitors and RIPA. The supernatant was then obtained by centrifugation at 12,000*g* for 10 min. Subsequently, 10 μg of total protein was resolved in 10% SDS-PAGE and transferred to a polyvinylidene fluoride membrane. The following primary antibodies were used for immunoblotting: anti-peroxisome proliferator-activated receptor-γ (CST), anti-CEBPβ (CST), anti-FABP4 (CST), anti-FASN (CST), anti-Bcl-2 (ZEN-BIOSCIENCE), anti-Bim (ZEN-BIOSCIENCE), anti-actin (Proteintech), anti-Cleaved Caspase 3 (Proteintech), anti-Cleaved Caspase 9 (Proteintech, China), anti-IGF2BP2 (Thermo Fisher Scientific). Images were captured using an iBright imaging system (Thermo Fisher Scientific) and the electrochemiluminescence method. Images were analyzed in grayscale using ImageJ (https://imagej.net/ij/) software on GitHub.

### Cell culture and transfection

The 3T3-L1 cells were purchased from Cell Resource Center, Shanghai Academy of Life Sciences, Chinese Academy of Sciences and grown at 37 °C in 5% CO2 in Dulbecco's modified Eagle's medium (DMEM) containing 10% fetal bovine serum (FBS), 100 UI mL−1 penicillin, and 100 μg mL−1 streptomycin. Adipogenic differentiation was induced when cells reached 80% confluence. Cells were grown in induction medium (DMEM containing 10% FBS, 0.5 μm isobutylmethylxanthine, 1 μm dexamethasone, 0.5 μm rosiglitazone, and 20 nm insulin) for 2 to 3 days and then switched to differentiation medium (DMEM containing 10% FBS and 20 nm insulin). The siRNAs were transfected using Lipofectamine 3000 (Invitrogen). The sequences of the siRNAs used in this study: (*siCtrl*: UUCUCCGAACGUGUCACGUTT; *siBim-1*: GGCACUUGCUACCAUUCUACC; *siBim-2*: UAGAAUGGUAGCAAGUGCCUG).

### Flow cytometry

HF-treated cells were collected into centrifuge tubes using trypsin (Solarbio), washed with PBS, and resuspend the cells with basal medium. The number of cells was controlled at 1∗10^6^. Cells were stained with Annexin V/PI (Thermo Fisher Scientific) for analysis of apoptosis by flow cytometry.

### Virtual screening and molecular docking

Metabolically relevant protein structures were downloaded from the Protein Data Bank database and preprocessed using PyMOL (https://pymol.org/) and MGLtools (https://ccsb.scripps.edu/mgltools/), including ligand removal and export to pdbqt format. The optimal activity pockets of the protein molecules were predicted and set using AutoDockTools.The structures of HF were minimized. Batch virtual screening was performed using Vina to evaluate their potential binding capacity based on ligand and receptor binding modes, scoring, and potentiality. Docked structures were visualized in 3D and 2D using PyMOL and ligplot+ (https://www.ebi.ac.uk/thornton-srv/software/LigPlus/).

### Molecular dynamics simulations

Prepare the topology and coordinate files of the molecular system, the protein structure of IGF2BP2 was obtained from the AlphaFold2 database. Select the GROMACS (https://www.gromacs.org) classical small molecule force field spc. The simulation box was constructed using the editconf tool with a box boundary of 1 Å. After adding the water model and ions, the simulation was minimized by using the mdrun tool. Conformational constraints were removed using the genrestr tool to minimize the system energy. NVT (constant volume constant temperature) and NPT (constant pressure constant temperature) simulations were performed using mdrun for thermal equilibrium of the system. Kinetic simulations were then run through mdrun for 200 ns to record the molecular motion and interactions in time. Finally, trajectory analysis, visualization, structural analysis, and kinetic parameter calculations are carried out, and the data are processed and visualized using tools and scripts provided by GROMACS.

### CETSA and isothermal dose-response fingerprint (ITDRF_CETSA_)

Subsequently, 3T3-L1 cells were cultured to 90% density and then treated with RIPA into a cell lysate ready for experiments. HF is added to the cell lysate at a final concentration of 10 μM. The cell lysate was placed on a series of hot plates at different temperatures for heat treatment. The lysed cells were centrifuged to isolate the soluble proteins in the supernatant. The amount of IGF2BP2 protein after treatment at different temperatures was detected by Western blot to determine the thermal stability of the protein. The thermal stability and binding affinity of the proteins were assessed based on the changes in protein content at different temperatures. Isothermal dose-response fingerprint CETSA was performed according to the optimal temperature obtained. The difference with CETSA was that HF was added to the cell suspension at final concentrations of 10^−3^, 10^−2^, 10^−1^, 10^0^,10^1^, 10^2^, and 10^3^ μM and heated at 68 °C.

### Construction of SS-m^6^A and Bim mRNA plasmids

SS-m^6^A was synthesized based on previous studies on IGF2BP2. WT and MUT plasmids containing m^6^A modification sequences that are common to different transcripts of Bim were constructed through the NCBI database and SRAMP database.

### Methylated RNA immunoprecipitation

Extracted RNA was fragmented using fragmentation buffer (GenSeq). The immunomagnetic beads were incubated with m^6^A antibody and immunoglobulin G antibody and subsequently mixed with the fragmented RNA. The immunomagnetic beads were washed to remove nonspecific binding RNA and release the captured m^6^A-modified RNA. Enriched methylated RNA was purified and subjected to qPCR. Primers used to detect Bim expression forward: TTGTTTCCCTTGCCTCCTCG and reverse: GGCTGCAATTGTCCACCTTC.

### RNA pull-down

RNA pull-down assays were performed in accordance with the Pierce Magnetic RNA‒ Protein Pull-down Kit protocol. Biotin-labeled ss-Bim m^6^A RNA and ss-Bim mutant RNA probes were synthesized by Accurate Biology, while the control group contained only protein lysate. Total protein was extracted from each group at a desired protein concentration greater than 2 mg/ml. IGF2BP2 was pulled according to a standard protocol, and expression levels were confirmed by Western blotting. ss-Bim m^6^A group sequence RNA: UGUGA CAGAG AAGGU GG (m^6^A) CA AUUGC AGCCU GCUGA; ss-Bim MUT group sequence RNA: UGUGA CAGAG AAGGU GGGCA AUUGC AGCCU GCUGA.

### Statistical analysis

All experiments were replicated at least three times. All data were obtained from at least three independent replicate experiments and were presented as mean ± standard deviation (SD). Data were statistically analyzed by GraphPad Prism version 9.0 (GraphPad Software). Comparison between two groups was analyzed by unpaired Student’s *t* test. Multiple comparisons were made using one-way ANOVA and Two-way ANOVA. The differences were statistically significant when *p* < 0.05.

## Data availability

Data from this study are either included in this article or can be requested directly from the corresponding author. In addition, the sequence data are available on the Gene Expression Omnibus (GEO) under the accession number GSE270326.

## Supporting information

This article contains supporting information.

## Conflict of interest

The authors declare that they have no conflicts of interest with the contents of the article.

## Supporting information

Supplementary materials

Visualization of molecular dynamics simulation.mp4
