# Supplementary material for: Hinokiflavone resists HFD-induced obesity by promoting apoptosis in an IGF2BP2-mediated Bim m6A modification dependent manner
Source: J Biol Chem. 2024 Aug 29;300(9):107721. doi: 10.1016/j.jbc.2024.107721 (PMC11465056; doi:10.1016/j.jbc.2024.107721)
Supplement: Supplementary materials [file mmc1.docx]

**Supplementary materials**

**Supplementary Figure 1. Tissue weight changes in HF-IP HFD mice. (A)** Body size changes in mice. **(B)** Changes in muscle and liver weight, (n=7). GAS: Gastrocnemius; TA: Tibialis Anterior; SOL: Soleus; EDL: Extensor Digitorum Longus.

**Supplementary Figure 2. The effect of HF in proliferating 3T3-L1 cells. (A)** The effect of different HF concentrations. The scale bars represent 100 μm. **(B)** CCK-8 assay to measure cell viability, (n=3), **p* < 0.05, ****p* < 0.001 by One Way ANOVA between paired groups. **(C)** EDU immunofluorescence assay to detect cell proliferation. The scale bars represent 200 μm. **(D)** Statistical analysis of cell proliferation, (n=3). **(E)** Flow cytometry analysis to detect cell apoptosis by annexin V binding and propidium iodide (PI) uptake.

**Supplementary Figure 3. Effect of Bim knockout on apoptosis and lipogenesis genes.**

**Supplementary Figure 4. Design of Bim mRNA m^6^A modification site and the binding ability between Bim and IGF2BP2. (A)** Presentation of different transcripts of Bim. **(B)** Prediction of shared m^6^A modification sites among different transcripts of Bim. **(C)** RNA pulldown experiment to verify the binding ability between Bim and IGF2BP2.

**Supplementary Movie 1. Visualization of molecular dynamics simulations.**
